# Development and implementation of a new model for research support for municipal healthcare—a qualitative study

**DOI:** 10.3389/frhs.2025.1686425

**Published:** 2025-12-12

**Authors:** Maria Bjerk, Oddvar Førland, Lars Bergersen, Lars Jørun Langøien, Lillebeth Larun

**Affiliations:** 1 Division of Health Services, Norwegian Institute of Public Health, Oslo, Norway; 2 Centre for Care Research, Western Norway University of Applied Sciences, Bergen, Norway; 3 Bergen Kommune, Bergen, Norway

**Keywords:** evidence-based practice, informed decisions, municipal healthcare, decision-making, research support

## Abstract

**Background:**

Evidence-based practice means making decisions based on evidence which takes account of experiences, values and preferences of employees and users. Fragmentation of services, technological limitations, lack of workforce, cultural resistance, resource constraints and distance between academia and practice can make the utilisation of evidence in health and social care services challenging. This study aimed to provide new insights into the development and implementation of a model for research support for decision-makers in municipal healthcare.

**Methods:**

We used a qualitative design to explore stakeholders’ experiences with development and implementation of the model for research support. We included minutes from several meetings and evaluation forms from the participating municipalities, ranging from the start of the project in January 2021 to the end of the project in January 2024. We conducted a thematic analysis, and the textual data were coded into categories and mapped according to the constructs of the consolidated framework for implementation (CFIR).

**Results:**

The stakeholders in the municipalities expressed need for a support model to apply research in prioritising, planning and decision making. There were barriers to implementing the model due to complex and broad research questions. The researchers needed to navigate between methodological thoroughness and practical usability. The participants from the municipalities reported lack of structure, funding, competence and incentives to apply the evidence. They also struggled with dissemination and implementation of the results from the research summaries. Facilitating factors were political and administrative commitment, availability of research findings in plain language, a learning-by-doing approach through meetings and seminars working on real-world municipal challenges, and a structured collaboration between municipality employees and academics.

**Conclusions:**

The study indicates that research support for decision-makers in the application of systematic reviews can be useful for evidence-based decision-making in municipal healthcare. However, implementing the model is resource-demanding, considering the use of time and personnel, both from the municipalities' and research institutions' point of view. Future research is needed to assess the effectiveness of the research-based support model towards better decision-making in municipalities and improvedpatient care.

## Background

The availability and use of evidence in health and social care faces several challenges in Western welfare states, due to fragmentation of services and resource constraints (1). In Norwegian municipal healthcare, lack of organisational resources and support, and a limited number of personnel with an advanced level of education, are important challenges impacting the use and implementation of research-based knowledge (2, 3). Thus, developing a model to support municipalities in employing research in decision-making to improve local policy and practice is important. Research-based knowledge is a key element in the concept of evidence-based practice, which means making decisions on the basis of systematically gathered research-based knowledge; employees' experience-based knowledge; and users' experiences, values, and preferences (4, 5). In a revised model focusing on decisions in public health and incorporating the transdisciplinary perspective, the model includes the best available research evidence; the client's or population's characteristics; and the needs, values, preferences and, finally, resources, which are all placed in a setting of environment and organisational context (6). The different types of knowledge complement each other and can inform professionals' and leaders' decisions and practices. Professional judgment is essential when applying general guidelines and research recommendations to newer or specific contexts, as well as when combining research-based knowledge with employees' and users' experiences. Such professional judgement is a prerequisite for individualised treatment (7).

Evidence-based decisions are important in clinical encounters and at the system level. However, a discrepancy in what is known and what is done, leading to repeating interventions showing little or no effect, is a challenge (8). Other important challenges are preference for simple solutions to complex problems, skipping the assessment of how to achieve changes, and limited sharing of knowledge between professions (8, 9). Several models of decision-making tools aiming at solving these challenges and promoting evidence-based decisions have been evaluated (10–12). The GRADE-evidence-to-decision framework aims to help decision makers use reviews in a structured and transparent way (11). This framework has mostly been employed by international organisations such as the World Health Organisation (WHO) for developing guidelines. Another decision-making tool used in hospital care is the mini-HTA (Health Technology Assessment), a tool to support evidence-based decision-making when planning to introduce new health technologies into clinical practice (12, 13). In Denmark, the Danish National Board of Health introduced mini-HTA as a management and decision support tool for the municipality, and they discovered several barriers to implementation, such as a lack of education among employees and managers (12). In the Nordic countries, municipalities play an important role in the healthcare system. Municipalities are responsible for providing all or parts of primary healthcare services as well as prevention, rehabilitation and health promotion interventions (14). To provide the best possible healthcare, it is necessary to enable municipalities to make evidence-informed prioritisations and actions. However, there is limited research on municipal healthcare as well as limited use of research within municipal decision-making (15). In line with this, a white paper in Norway highlighted the importance of developing a structure for an evidence-based support system for 357 municipal healthcare services in Norway (16). For these municipalities, prioritising services which deliver greatest benefit and which are targeted for those most in need, is demanding, as opportunities and demands often exceed available resources (17). Thus, healthcare personnel, administrative leaders and politicians need usable tools for prioritisation and a research base to be able to further develop municipal services. Despite the limited research relevant to the fields within municipal healthcare, the volume of research literature is increasing. However, most of the literature is not accessible to municipal employees or managers since municipalities have scarce access to subscription journals/databases (18). Municipal healthcare have few resources, capacity and structured methods to ensure that new research is applied. Thus, our study aims to provide knowledge regarding the development and implementation of a model for research support for decision-makers in municipal health care. The research question was: what experiences do municipal stakeholders have with a new model for research support?

### Theoretical framework

Knowledge translation refers to a variety of activities designed to facilitate the application of research findings, ultimately promoting evidence-based practice (19). It is a dynamic and iterative process that involves the synthesis, dissemination, exchange, and ethically sound application of scientific knowledge to improve health outcomes, provide more effective health services and products, and strengthen healthcare systems. This process helps to effectively integrate research results into practice, bridging the gap between what is scientifically known and what is done in healthcare settings (20). Knowledge translation often requires significant adaptation to local contexts, which can be complex and resource-intensive. The one-size-fits-all approach is rarely effective, and tailoring strategies to specific settings can be challenging but necessary (21). Effective knowledge translation relies on strong collaboration between researchers, practitioners, and policymakers. However, fostering these relationships can be difficult because of differing priorities, communication barriers, and power dynamics (22).

**Figure 1:**
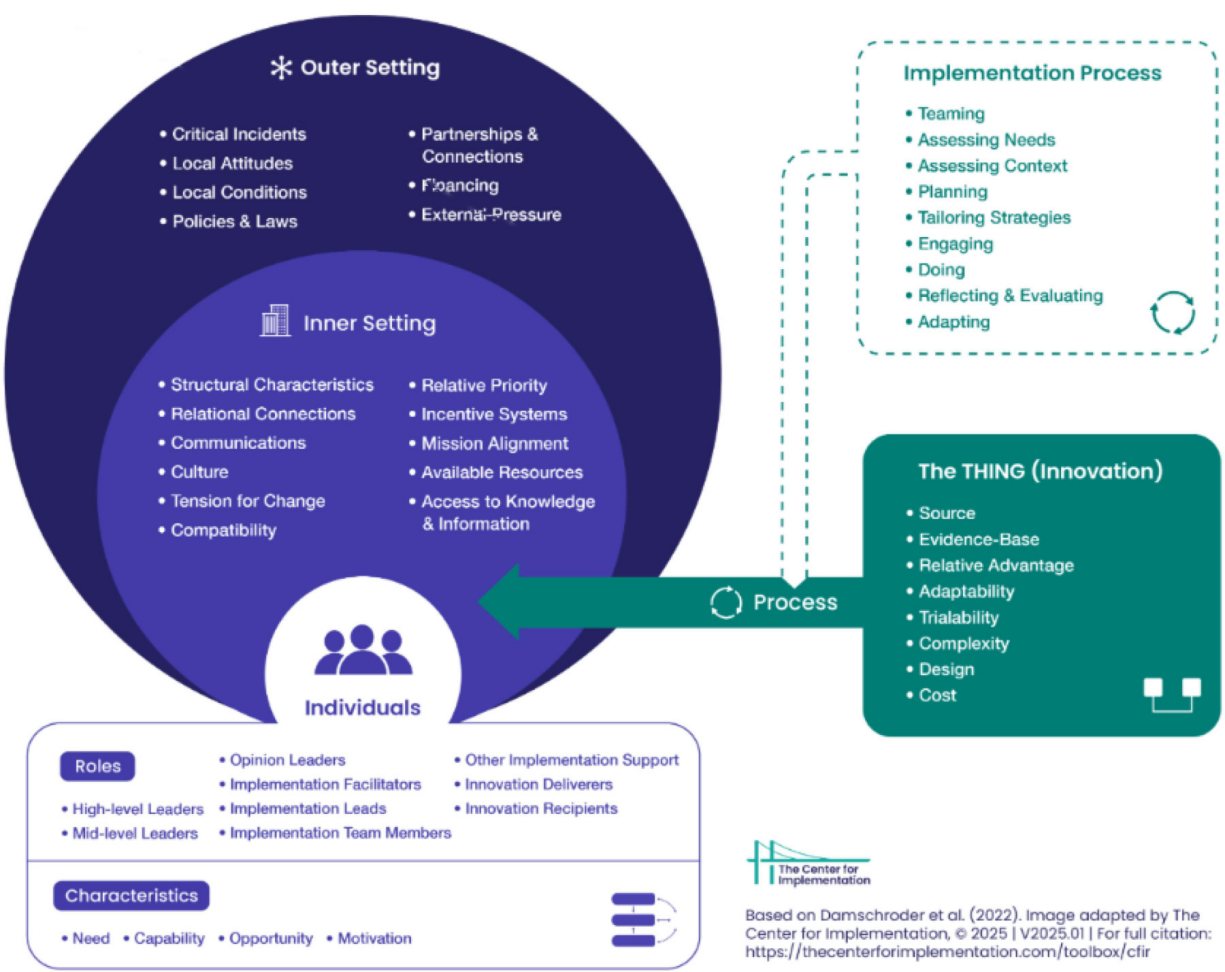
The consolidated framework for implementation research (CFIR) (23).

To assess the development of the research support model described below, we applied the consolidated framework for implementation research (CFIR) in the analysis process to structure the data and identify possible facilitators and barriers (Figure 1) (23). CFIR has integrated constructs from multiple existing implementation theories, identifying what works where and why and across multiple contexts (23). The model organises the barriers and facilitators into five overarching domains: (1) intervention characteristics, referring to attributes of the intervention itself, such as complexity and adaptability; (2) outer setting, including external influences such as policies, incentives, and patient needs; (3) inner setting, including organisational factors such as culture, leadership engagement, and readiness for change; (4) characteristics of individuals, encompassing the knowledge, beliefs, and self-efficacy of those involved in the implementation process; and (5) the implementation process, which involves planning, engaging, executing, and reflecting on implementation activities (24).

CFIR is a determinant framework that can be used in both the planning phase and the evaluation phase (23). This framework has been used in multiple settings and for multiple purposes, such as for guideline implementation in hospital-based nursing practices (25) and for guiding rapid evaluations of an implementation process in primary care (26). Previous research underlines the benefit of using CFIR to guide an efficient and rigorous analysis of rich qualitative data. It also highlights the challenges associated withmissing domains such as characteristics of health care systems (27) and the consideration of stakeholder aims during implementation (25).

## Material and methods

### Study design

We used a qualitative design to explore stakeholders' experiences during the development and implementation of a model to support the use of research in decision-making in municipalities. Stakeholders were defined as those involved in developing the model and those receiving the research-based support. The study reporting follows the Consolidated Criteria for Reporting Qualitative Research checklist (28) (see Supplementary File) and the Standards for Reporting Qualitative Research (29).

### Stakeholders and setting

This project was carried out from 2021 to 2024 and was administered by Bergen municipality, which leads a regional network for cooperation between municipalities and research organisations called the “Kunnskapskommunen Helse Omsorg Vest” (The Knowledge Municipality in Health and Care in West). The stakeholders were employees and decision-makers from municipalities recruited in Western Norway and researchers and librarians from the Norwegian Institute of Public Health (NIPH) and the Western Norway University of Applied Sciences (HVL). The number of employees from municipalities varied between two and five, and majority of them were managers, health personnel with professional responsibility, advisors and economists. We recruited six researchers and two librarians from NIPH and three researchers and four librarians from HVL. Two advisors from Bergen municipality also participated. Additionally, one manager each from NIPH and HVL and two advisors from the Norwegian Association of Local and Regional Authorities (KS) participated in a reference group.

### The model developed

**Figure 2:**
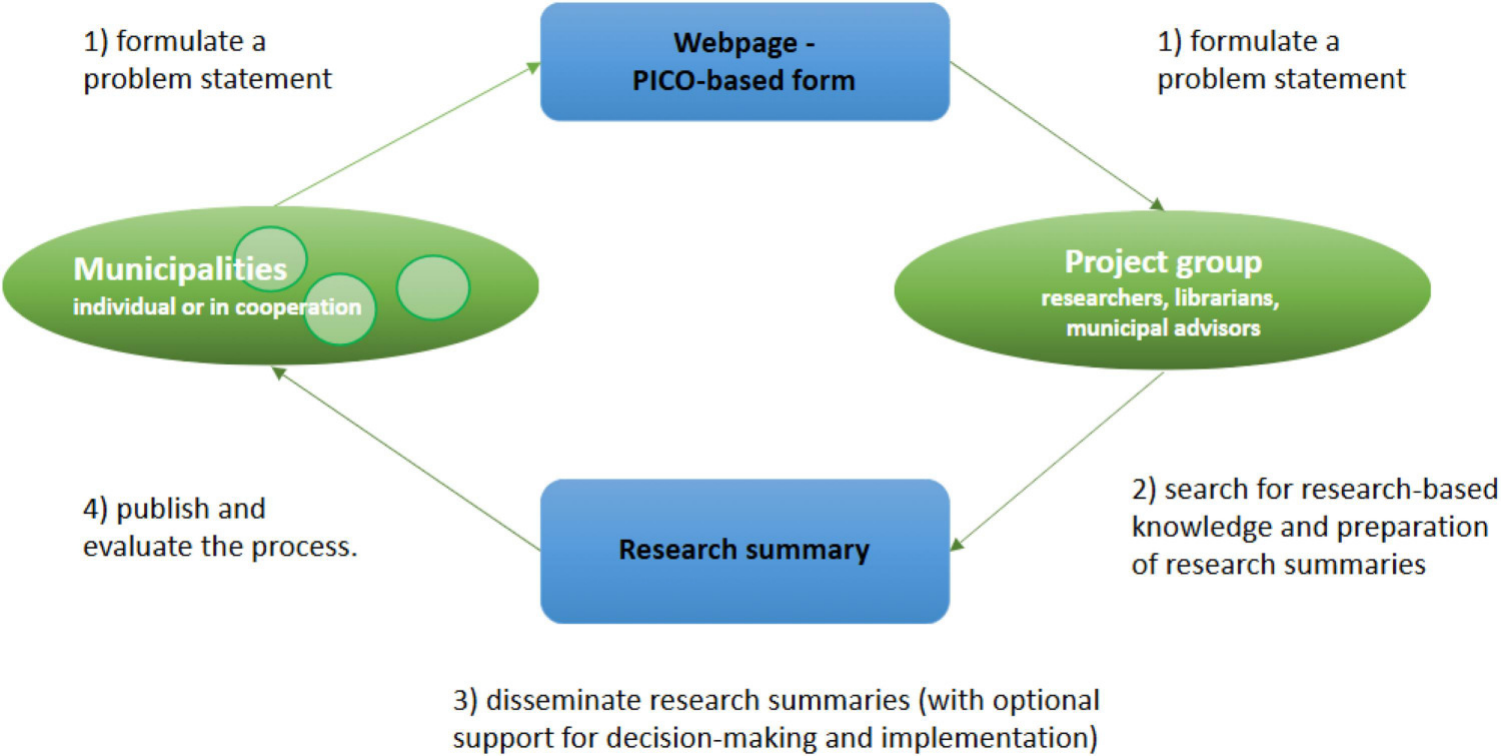
Structure of research-based support for municipalities, including step 1–4.

In a pilot project conducted in 2019–2020, a structure was developed together with the municipalities in “Kunnskapskommunen” (The Knowledge Municipality), testingrequesting research-based knowledge and identifying and disseminating already summarised research. The purpose of the structure was to strengthen different processes for support: from the identification and formulation of needs for research-based knowledge to the preparation for the application and implementation of research knowledge in a municipal evidence-based practice. An overview of the structure is shown in Figure 2.

For the study described in this paper, we built on experiences from the pilot project conducted in 2019–2020. We focused on the phase of producing research summaries, starting with formulation of questions to publication and evaluation of the process. The steps in the model are: 1) formulate a problem statement; 2) search for research-based knowledge and preparation of research summaries; 3) disseminate research summaries with guidance in decision support processes and on the implementation of research knowledge in services; and 4) publish and evaluate the process.

#### Step 1: Formulation of a problem statement

The core of this model was that employees and managers in the municipalities themselves were aware of their need for research-based knowledge. A form was created on a regional website where employees in municipalities could develop a problem statement. These statements could be related to professional, administrative or political decision-making processes, such as municipal plans or the introduction of new health interventions. The main questions in the form were based on PICO (population, intervention, comparison, and outcome), which isa tool that provides structure and clarifies the question for literature search and selection criteria (30).

#### Step 2: Search for research-based knowledge and preparation of research summaries

The model included four to five meetings between the researchers, the librarian and the municipality group. The first three meetings focused on goals and PICO, a literature review, and the presentation of findings. Before the first meeting, we performed an initial literature search in databases such as Epistemonikos and Cochrane to obtain an overview of the research field. In the first meeting, we clarified the problem statement. Following the first meeting, the librarian performed a systematic search, and two researchers independently screened the literature. The researchers identified relevant systematic reviews that met the selection criteria and quality standards. In the second meeting, the researchers presented preliminary findings from the literature search, and the group discussed which of these findings were most relevant. Following this meeting, the researchers assessed the methodological quality and wrote a research summary. Additionally, in some cases, we conducted GRADE (Grading of Recommendations Assessment, Development and Evaluation) as a tool to evaluate the certaintyc of the evidence (31). By using GRADE, we evaluated risk of bias, inconsistency, indirectness, imprecision and publication bias and wrote the results using standard GRADE formulations. If multiple reviews addressed the issue, we assessed the need to create multiple summaries. The research summary was sent for internal peer review to two researchers.

#### Step 3: dissemination of research summaries

The third meeting covered the dissemination of the research summary and its application to practice. We presented the research-based results, the certainty of the evidence and discussed the dissemination of the research. The purpose of meetings four and five was to provide support for applying the research summary, either on its own or in combination with other knowledge for decision-making support. How the research summary was used largely depended on the nature and purpose of the project in the municipality. Research summary was used in various contexts, such as in political plans, in internal seminars, in videos for competence development, as evidence in an evidence-to-decision (EtD) process or as part of a mini-Health Technology Assessment (mini-HTA). When the research summary was applied in a EtD or mini-HTA, it was included in conjunction with information on resource use, economic benefits and information on organisational issues and ethics. Table 1 presents examples of topics, research summary or summaries made and the stakeholders application of them.

**Table 1:** Examples of topics, research summaries and applications.

Topic	Research summary/summaries	Application
Welfare technology for people with developmental disabilities	1) Mobile technology can support people with disabilities in their daily lives. 2) Assistive technology in the workplace for people with developmental disabilities. 3) Technological support for grocery shopping for pupils and students with developmental disabilities.	Educational videos for employees working with people with disabilities
Recruitment of health care professionals with a bachelor's degree in health and care services	Measures to recruit and retain healthcare personnel	Seminar for managers making decisions on interventions for recruitment of personnel
Loneliness among older adults	1) Measures to prevent loneliness among older adults 2) Common features of effective intervention preventing loneliness among older adults	Policy and strategy plan for public health work in one municipality
Digital home monitoring in home care services	1) Remote monitoring for people with COPD 2) Digital home monitoring of patients with diabetes and/or high blood pressure	Mini-HTA employed as a basis for decision-making and planning of the implementation of home monitoring in home care

#### Step 4: Publication and evaluation

The research summaries were published on the webpages of The Norwegian Institute of Public Health (Folkehelseinstituttet), Norwegian Health Library (Helsebiblioteket), The national Care Library (Omsorgsbiblioteket) and the Knowledge Municipality Health and Care West (Kunnskapskommunen HelseOmsorg Vest). Following the publication of the research summary, the municipalities completed an evaluation form and participated in an evaluation meeting.

### Data collection and management

To enable a wide range of input, we included minutes from different meetings and evaluation forms from the participating municipalities, ranging from the start of the project in January 2021 to the end of the project in January 2024. Table 2 below presents the amount of data from the meeting minutes.

**Table 2:** Overview of meeting minutes (*n*).

Year	Project management: Norwegian Institute of Public Health, Western Norway University of Applied Sciences and Bergen Municipality	Research method: Norwegian Institute of Public Health and Western Norway University of Applied Sciences	External feedback: Reference group
2021	*n* = 37	*n* = 8	*n* = 8
2022	*n* = 38	*n* = 9	*n* = 17
2023	*n* = 28	*n* = 9	*n* = 19

The research group at the Norwegian Institute of Public Health, also including two advisors from Bergen municipality (“Kunnskapskommunen”), had weekly meetings as well as regular collaboration meetings with researchers and librarians at the Western Norway University of Applied Sciences. The topics at both meetings were issues related to methodology, such as the literature search, selection of literature, tools for screening, quality appraisal, writing of the research summary and dissemination of the research summaries. In addition, we had meetings with the reference group consisting of managers from the Norwegian Institute of Public Health and the Western Norway University of Applied Sciences, advisors from Bergen municipality and two representatives from the Norwegian Association of Local and Regional Authorities. Topics of these meetings encompassed issues at the system level, such as funding, anchoring within the different regional or national health service authorities and sustaining the projects within the municipalities.

Another data source for the analysis was the evaluation forms from 16 projects completed by the municipalities. In the evaluation forms, we sought feedback from the participants in the municipalities regarding their involvement in the initial phase, contact during the process, time usage, usefulness, and support for dissemination.

### Data analysis

**Figure 3:**
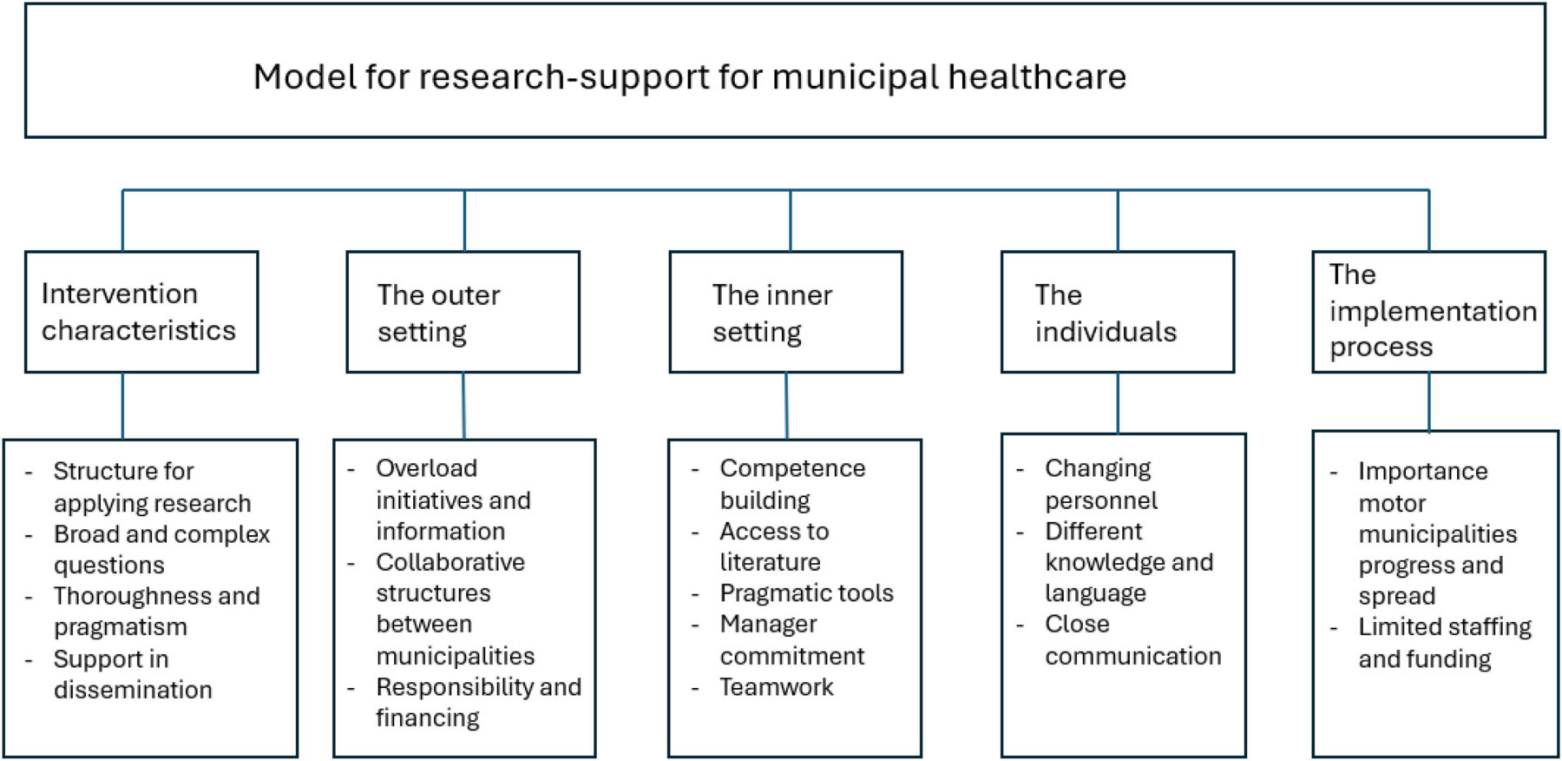
Analysis process, including categories in CFIR and related codes.

The data included documents, and we used the thematic analysis approach of Braun and Clark to analyse it (32). First, data from the meeting minutes and evaluation forms were systemised chronologically in one document. Second, two researchers independently read the material thoroughly, taking notes and looking for themes across the material regarding experiences with the model. All authors in collaboration discussed emerging patterns and themes and checked it against the material. The textual data was further coded into categories and mapped according to the constructs of the CFIR framework: the innovation, the outer and inner setting, the individuals and the implementation process (33). The innovation was defined as the model for research support, the outer setting was defined as surrounding organisations such as the Norwegian Association of Local and Regional Authorities (KS), the Norwegian Ministry of Health and the Norwegian Directorate of Health and the inner setting was defined as the municipalities and research institutions. The individuals included the innovation deliverers and the innovation recipients, and the implementation process included reflection, evaluation and tailoring strategies. Following the structuring of the data according to the CFIR, the themes and categories were again checked against the raw data to ensure that they reflected the data. Figure 3 below provides an overview of the analysis process, presenting codes according to the themes in CFIR.

## Results

### The innovation

The general feedback from the participating municipalities indicated a need for a support model with summarised research to prioritise and plan health care measures or new interventions. The topics and questions of interest reported by the municipalities were often used when drafting administrative plans or deciding on new interventions. They expressed a need for a structured way to formulate questions, identify relevant research, summarise and present evidence-based information in a way that the stakeholders found useful. The following quote from one of the municipal advisors reflects this:

“There is little research within the municipal healthcare services. Broad topics and the use of various types of design are common. We are likely to experience many times that the quality of the research has not reached moderate or high. What do we do then?”

A reported challenge was that typical research questions for the municipalities were often broad, concerning either the intervention or the population. Stakeholders found the PICO framework from evidence-based practice helpful. Given the complexity of municipal challenges, the project group often moved beyond effect studies to include qualitative research to broaden the perspective and to better answer the question of the municipality. Nevertheless, the model was relevant, as described byone of the researchers:

“The project's innovation is a cross-municipal organisational model for the best possible knowledge-based service delivery, which aims to lead to increased accuracy and improved quality. The model allows municipalities to jointly prioritise their knowledge needs ”

Researchers and librarians noted the challenge of balancing thoroughness and pragmatism in the methodology. A systematic, collaborative approach to select, assess and summarise the systematic reviews was seen as important to ensure transparency and reliability. Both researchers and municipalities experienced challenges in applying GRADE to transform research into practical outcomes. Many reviews relevant to municipalities included small-scale studies with different study designs, often resulting in a low certainty of evidence. A quote from one of the researchers reflects this:

“The challenge is that the quality of the reviews is not very good. There is a lack of protocols, several studies have not described the search strategy, and many have not assessed the quality of the included studies. Several of the reviews are well written regarding the topic but are unclear in regard to the methodology. The challenge is then how strict we should be when selecting a systematic review.”

A two-page summary of each review, including context information, was created on the basis of Cochrane brief summaries. The researchers helped develop templates and content descriptions, and the summaries were well received. The final output of the process was evidence summaries, and the municipalities highlighted the need for support through dissemination. Despite this, the municipalities still struggled to implement the findings. One of the librarians described it as follows:

“It is challenging for the municipality to find ways to utilise the research. We must be patient and provide support along the way. It is somewhat easier to use the research when the goal is planning work.”

### The outer setting

The Norwegian government requires municipalities to work evidence based. The Norwegian Association of Local and Regional Authorities and the Norwegian Institute of Public Health initially developed models in parallel but merged efforts to create and implement a model for research-based decision-making for municipalities. A collaboration with academia was also established. Municipalities reported lacking structure, funding, competent personnel and sometimes motivation or incentives to employ evidence-based knowledge in decision-making and development of their services. Smaller municipalities found this particularly difficult. Municipal administration sometimes appeared unclear about evidence-based decision-making, which could affect its application. One of the municipal advisors described this as follows:

“The model must be anchored at the political level, even though this sometimes involves shifting responsibility to the municipality. The question is whether there should be state-coordinated financing. There are guidelines today, but as of today, there are no structures to incorporate those guidelines into.”

Municipal and research representatives saw value in a structured approach to applying research, where prioritisation could be made centrally, or in collaboration with other municipalities, given that challenges are often the same. Despite the recognised need for evidence in applying and implementing evidence-based practice, the responsibility for this remains unclear. Is it the municipalities, academia or central political administration that should take the lead? One of the members of the reference group reflected on this:

“We need an instance that is a driving force and propels the work forward. There are many knowledge actors, and it is difficult to collaborate effectively. It is important that we speak with one voice since everyone wants to advocate for their issues. We must have a unifying voice.”

Municipalities reported feeling overwhelmed by information, and even though they appreciated autonomy, it was necessary to navigate between evidence and other types of information, such as guidelines, and it was not always clear whether such information was based on research. One proposed solution involved having a structure or webpage that coordinates information and evidence to improve health services. Today, several municipalities, researchers and competence centres are duplicating work. One of the librarians stated:

“It is important to ensure that too many resources are not used by doing a lot of duplicate work. There must be platforms to share both knowledge and to report knowledge needs.”

### The inner setting

Building competence in applying research-based evidence within municipal healthcare was a key objective of the model. The municipalities reported that they needed a better understanding of research methods, evidence-based practices, and how to evaluate and apply research in their work. A challenge for the municipality's healthcare was the limited access to research literature and librarians. One of the municipal advisors described:

“Need knowledge in order to make use of the research and to understand where it comes from. Both general knowledge of methods and evidence-based practice, and what it actually involves, are more concrete. What does one do, and how can research-based knowledge be applied?”

The methods for identifying problems, conducting searches, and selecting and summarising evidence from the reviews were time-consuming. Finding or developing tools that were adequate, easy to use and understandable in the municipal setting was challenging but rewarding. A quote from one of the municipal advisors reflected this:

“It doesn't matter that the processes have dragged out. Has then had time to digest the information. The process has created awareness about research and the use of research in practice.”

Another important factor for implementation was manager commitment. Such engagement was necessary to ensure that the local knowledge of the organisation and resources was considered and that research-based information was used in decision-making. One of the municipal advisors reflected as follows:

“Researchers who provide knowledge summaries must give us a clear expectation that it will be anchored in the organisation. This is something we have worked on from the beginning. What will it be used for? Should the leaders participate in the meetings? How much of the process should they be involved in? It is important to ensure that the inputs are useful for more people and that it is not just one person who ends up with this.”

Manager commitment remained crucial in the final implementation phase. Several municipalities reported weak manager commitment or turnover among central team members, which made dissemination and implementation challenging. Support for municipalities in these processes was needed but often debated, as it falls outside the typical role of a researcher. One researcher stated:

“He states that the implementation process has stopped owing to a pressured leadership team and a coordinator on sick leave.”

A structure for collaboration between municipalities and researchers was developed during implementation. The municipalities and the researchers, including librarians, met three to five times over two months to define objectives, frame the problem, search, identify evidence and plan implementation. Researchers from different institutions held biweekly meetings to discuss practical issues such as screening tools and how to assess the risk of bias. The librarians met monthly to discuss matters such as different types of searches or search engines. Involving all stakeholders in the process helped to ensure the model's relevance and the integration of research findings into local practices. One of the librarians quoted:

“It was a good collaboration within the team. Nice that X and Y from the municipality were participating in all team meetings. It provides continuity. Very important to have input from the municipality all the way to ensure relevance.”

The teamwork was vital for continuity, with team members working flexibly on various commissions and supporting each other in sharing knowledge. Examples of this were reviewing summaries and working in pairs across institutions, with different research experience and methodological expertise. This collaboration was considered significant for the quality of the model. One reported challenge by the researchers was the limited recognition of this work, as the summaries were not published in scientific journals. Another challenge was the limited resources, which required strong motivation and interest from researchers. One of the researchers stated:

“Affiliation with research institutions – Should prioritise dissemination, but is this achievable? Some do not wish to spend time if it does not lead to scientific publishing—requires personal motivation and interest or investment from the institution.”

### The individuals

Cooperation between researchers and employees in municipalities was central to developing the model. Close collaboration with managers and advisors throughout each project ensured the ownership and relevance of the research summaries. One challenge was that the processes were vulnerable to sick leave or turnover. To ensure continuity, it was essential to include multiple stakeholders in each municipality, especially managers, to ensure leader commitment. A quote from one of the municipal advisors described this:

“The process was done to a small extent in collaboration [internally]. Ideally, this should have been done, but owing to many parallel work tasks, low capacity in the city council department, combined with COVID-19, only one advisor submitted the proposal. The manager was, however, informed. It would have been nice if they had gathered more and prioritised proposals, they wanted research on.”

The municipalities and academic partners included in the implementation had different knowledge of research and research methods. Several researchers had extensive experience within primary research but less experience in systematic reviews. Workshops, educational material and support systems were codeveloped in literature searches and assessments of methodological quality. We also used paired mentorship between researchers from different institutions. Clear communication and plain language were essential when working with municipal employees in seminars and meetings. A template for brief summaries of systematic reviews was developed, making the research more accessible. One municipal advisor stated:

“Incredibly good, close dialogue, asking about things along the way. Researchers are good at talking to practice. Just tell about challenges and difficulties and how they were navigated through them in a good way. Not important with a specific professional background, the language is understandable.”

### The implementation process

The implementation process faced both facilitating and challenging factors in the inner as well as the outer setting. The structure of the model and Bergen municipality's role as a “motor municipality” was important for sharing knowledge and identifying knowledge needs in the municipalities. Bergen municipality led the processes and included smaller municipalities within their regions, leveraging their greater resources and expertise to interpret and apply research. We also collaborated with a related regional project, holding regular meetings to share experiences and challenges and to align approaches to municipal commissions. Implementing the model within a collaborative structure facilitated the spread of information and the opportunity to provide feedback to the governmental offices on what was needed to scale-up the use of research in municipalities. One of their searchers described:

“The collaboration with the other project has been an educational experience. They work more with the start of the process – how to come up with research questions. It is something we can benefit from. It has also been nice to have them as participants in workshops in methods support to provide feedback”.

Limited staffing and funding in both the project group and municipalities posed challenges and required strategic decisions. Working mainly digitally saved resources and enabled collaboration with municipalities nationwide, regardless of location. The project team had to carefully evaluate each commission's depth, utility, practical value and whether it supported municipal decisions. These were requirements considered to take on a commission. In the early phase of the project, municipalities were encouraged to suggest commissions twice a year, but this led to too many suggestions, many lacking the necessary support from key decision-makers. We therefore switched to a rolling submission system for proposals, allowing more targeted and timelier prioritisation. Another issue was what type of research questions we should include. One of the researchers stated:

“New commissions. Should we continue to focus on a few commissions and follow them throughout the entire process? Or should we make an effort to obtain many commissions? Rather, get commissions and gain experience with decision-making and implementation.”

## Discussion

The findings in our study show that several factors, concerning both the innovation, the outer setting, the inner setting, the individuals and the implementation process, influenced the development and implementation of the model for research support in municipal healthcare. Both the outer and inner settings expressed a need for a support system for implementing research-based knowledge in municipal healthcare to provide resource-effective services of high-quality. National policies demand evidence-based practice, and municipalities struggle to absorb an ever-growing universe of research literature, where access to research evidence is often difficult. Challenges both concerning the implementation process, the inner and outer setting were lack of funding, structure, competence and incentives to apply evidence. There was a limited clarity in responsibility and roles of providing research support. Facilitating factors considering the implementation process, the innovation and inner setting was political and administrative commitment, communicating evidence in plain language, a learning-by-doing approach working on real-world challenges and a structured collaboration between municipalities and academia.

The close collaboration between the decision-makers in the municipalities and the academic partners was an overarching facilitator within the development and implementation process. The municipalities formulated research questions based on their challenges and researchers and municipal representatives collaborated to answer these questions, contributing different types of knowledge. Similar findings were provided by Betram and colleagues (34), who reported that local anchorage and collaboration between researchers and local governments increased the use of research knowledge. Involving end-users in both the development of the model and the research summaries has been crucial to transfer research-based evidence to local needs. The collaborative approach strengthened the ownership both within the municipalities and the health administration and could also be seen as a way to strengthen both system and human capacity in line with findings by Heimburg and colleagues (35). To ensure a strong partnership and trustworthiness, the researchers had comprehensive knowledge and experience in collaborating with municipalities. According to Hayes and colleagues, policymakers value researchers who can demonstrate competence, integrity and benevolence (36).

A key challenge was balancing between methodological rigor and pragmatic solutions to make the process and research summary applicable to decision-making in real-life municipal settings. In this setting, limited time, money, and staff made it necessary to decide when the work was “systematic enough”. These findings align with those of another study on the development of rapid reviews in health care, which revealed that the time needed to complete the review, the resources available and the complexity and sensitivity of the topic were important factors that affected the methods used (10). Another challenge was that in some cases, the research summaries did not provide all the information needed either to make a decision or plan an implementation. Therefore, some municipalities chose to conduct a mini-HTA where local knowledge on economic and organisational issues was included. This is in line with previous research which has reported that the knowledge‒practice gap can stem from a lack of concrete local knowledge, cost data and a shared understanding (37).

This study impacts practice and research at different levels. The model was feasible for both municipalities and research institutions. However, a drawback of the model is that it can be considered too resource-intensive to implement. There were extensive dialogic processes, and in the future, possibly faster AI-generated answers and recommendations will replace the more thorough background processes described in this model. At the same time, the collaboration between the researcher and municipalities led to a valuable exchange of knowledge across disciplines and fields of health care, and this process on its own could be valuable. Another evident challenge was the need to gather research-based information due to the overload and restricted access of the municipalities. Due to the digitalisation of work, there is a growing challenge of information overload, and organisational and technological interventions to reduce the amount of information presented are important (38). A national web resource disseminating relevant research summaries and other products of research could address this issue. With regard to future research, more knowledge on the effectiveness of the use of decision-making tools, including research evidence, is needed, as well as process evaluations of resource use and feasibility.

### Strengths and limitations

One strength of this study is the real-world setting i.e., the model was developed and implemented in the municipal healthcare services, with their challenges as a starting point. The model was funded, aiming at national implementation, and considerations were made to make the model sustainable. A broad range of stakeholders have contributed with data, from researchers to librarians and from academic institutions to municipality employees as advisors and managers. A limitation is that some meeting minutes were brief, and separate interviews with stakeholders might have added more depth to the data, but perhaps not the same comprehensiveness. Another limitation is the short follow-up at three to six months after publication, which in some cases was not long enough to evaluate the impact of the research summary on decision-making and implementation.

Author reflexivity is important. The project started with an idea in one municipality, driven by the need for research-informed decisions and poor access to relevant literature. One coauthor, with experience in both municipal administration and the Norwegian Research Council, was crucial in initiating the project on the basis of observations of ongoing challenges in applying research-based evidence to improve municipal services. The other authors have solid experience working with systematic reviews and primary research in the municipal healthcare setting. This background likely influenced the pragmatic approach to the model and made it more feasible, as did the stakeholders' input on what worked and what did not work.

## Conclusion

This study describes the development of a research support model for municipalities to support research-based decision-making, along with stakeholders' and decision-makers’ experiences. We offer a knowledge base for further development and implementation of such a model. The municipalities highlighted a need for a structure to apply research-based knowledge in planning and practice. A structured approach, including systematic reviews, five meetings, and a focus on dissemination, was found to be feasible for promoting evidence-based decisions in municipal healthcare, as an interaction between research-based knowledge and decision-makers' experience-based knowledge. Challenges remain, including limited research capacity, limited structures for knowledge sharing between research institutions and municipalities, and a lack of digital solutions to publish and gather research relevant to municipalities. Future studies should evaluate the model's impact on decision-making in municipalities and, ultimately, on better patient care.

## Data Availability

The raw data supporting the conclusions of this article will be made available by the authors, without undue reservation.
